# Mind the semantic gap: semantic efficiency in human computer interfaces

**DOI:** 10.3389/frai.2025.1451865

**Published:** 2025-03-26

**Authors:** James Horsley

**Affiliations:** Amazon Web Services, Seattle, WA, United States

**Keywords:** semantics, human computer interfaces (HCI), artificial intelligence, generative AI, semantic web, retrieval augmented generation, information theory

## Abstract

As we become increasingly dependent on technology in our daily lives, the usability of HCIs is a key driver of individual empowerment for us all. A primary focus of AI systems has been to make HCIs easier to use by identifying what users need and agentively taking over some of the cognitive work users would have otherwise performed, as such, they are becoming our delegates. To become effective and reliable delegates, AI agents need to understand all relevant situational semantic context surrounding a user’s need and how the tools of the HCI can be leveraged. Current ML systems have fundamental semantic gaps in bespoke human context, real-time world knowledge, and how those relate to HCI tooling. These challenges are difficult to close due factors such as privacy, continual learning, access to real-time context, and how deeply integrated the semantics are with in-context learning. As such, we need to research and explore new ways to safely capture, compactly model, and incrementally evolve semantics in ways that can efficiently integrate into how AI systems act on our behalf. This article presents a thought experiment called the Game of Delegation as a lens to view the effectiveness of delegation and the semantic efficiency with which the delegation was achieved.

## 1 Introduction: cognition in human computer interfaces (HCIs)

For millennia, humans have used tools and technology, taken in a broad sense, to make us more effective, productive, and increase the space of what’s possible. However, for most of that history, humans have been the driving cognitive force behind how those tools are created and employed to achieve our goals; we have only leveraged external agency and cognition when working with other life forms such as animals, plants, and micro-organisms. This status quo has been gradually changing over the last 80 years with the proliferation of information technology and the advancement of artificial intelligence, which have been driven by three key developments. First, the rise of the “information age” and the world-wide-web have provided a new scale of available data from which to train. Second, the steady increase in computational processing power and storage have provided hardware advancements and cloud-scale compute services. And third, recent advancements in the field of machine learning, particularly deep learning and large-language models.

Not only have the cognitive capabilities of our technologies increased, so too has our reliance on these capabilities as we have become increasingly dependent on technology in our daily lives. Technology impacts the everyday tasks of an individual’s shopping, traveling, medicine, finance, entertainment, home automation, communication, and community. Applying a wider lens, these developments are increasingly impacting the security and stability of nations as more governments employ them across daily operations, space exploration, climate analysis, and geopolitics.

As our bridge to technology, HCIs are a key driver of human productivity that can delight and empower us through ease of use, or frustrate and disempower us with endless cognitive friction. A “perfect” HCI would know exactly what the user needs and then act to meet those needs with minimal user effort. We can encode this as a guiding principle for intelligent HCIs

The principle of user need

*Give users what they need, when and where they need it*

The principle of user need suggests an HCI should understand (1) who the user is, (2) what the user needs, (3) what actions and tools are available in the HCI, (4) what actions the HCI agent should perform to meet the user’s needs, and (5) when and where the HCI should perform those actions. Current HCIs are far from achieving this ideal, they present users with tools and features but users must figure out how to discover, invoke, and combine those tools to achieve their goal, placing the cognitive burden almost fully upon the user. Traditionally, the cognition employed to make HCIs easier to use is the offline, up-front cognition performed by the humans who crafted the HCIs and associated tools. The HCI designers anticipate their users’ needs and create experiences that minimize user effort. However, since this work is performed ahead-of-time, the more complex the user’s job-to-be-done (Christensen et al., 2016) or more variability there is in how users can use the HCI, the higher the cognitive burden placed upon users.

Modern AI offers an alternate path by injecting dynamic and contextually informed cognitive capabilities into our HCIs. HCIs with intelligent agency (HCI agents) can not only reduce cognitive friction, they can also perform useful cognitive work on behalf of users, making technology dramatically easier to use. The word “useful” is an important qualifier as adding intelligence to HCIs is not itself the goal, the goal is to reduce the amount of cognitive work users need to perform to achieve their goals. Adding unhelpful intelligence can increase friction and frustration for users, particularly if the HCI agent performs work that the user has to “undo” or overcome to accomplish their actual goal. Adding intelligence can also increase user expectations for the HCI which, if unmet, further increases the chances and magnitude of user frustration.

## 2 Cognitive work

In this article we’ll define cognitive work as the amount of cognitive effort expended by an agent as part of achieving a goal, regardless of whether the agent is biological or artificial. Cognitive work captures that, in addition to any physical exertion required to complete a task, agents must expend time and energy on figuring out what they should do and how to do it. This is firstly achieved through activities such as observation, learning, and reasoning, which is heavily affected by the complexity of the act, and secondly by any cognitive attention and focus required to perform the action, which is particularly evident in high-precision, repetitive tasks. In this sense, cognitive work draws parallels to the concept of work in physics which, through the lens of energy transference and force, captures the energetic “cost” of action to a system.

Humans subjectively experience and intuitively communicate this framing of cognitive work, whereby we commonly refer to certain mental tasks leaving us feeling exhausted or drained. This distinction in mental effort is a key differentiator between Kahneman’s (2011) two modes of thinking, where system one is quick and lower-effort and system two is slower, more deliberate, and requires more effort to sustain. While the system one vs. system two framing distinguishes the difference in effort behind the modes of human thinking, the modes alone do not fully capture the “area under the curve” of cognitive effort, particularly in terms of (1) duration—for example performing sustained repetitive system one tasks, such as feeling tired after a long car drive, and (2) emotional duress or stress—for example taking a practice penalty-kick versus in the final moments of a high-stakes game.

For an HCI, cognitive work is the combined cognitive exertion expended by users and HCI agents in order to accomplish the users’ goals using the features (“tools”) provided by the HCI. Cognitive effort has intrinsic qualities such as the inherent complexity of the task and the ergonomics of the interface (cognitive friction). Cognitive effort also has user-relative qualities such as familiarity (having relevant priors), emotional engagement (what’s at stake), and general preferences (is this a task you enjoy). HCI creators have the most control over intrinsic HCI qualities around which the fields of UX design and research have created extensive frameworks that are captured as core principles and laws (Laws of UX, n.d.).

## 3 What semantic context do HCIs need?

Here we will define semantics as the truth and meaning of *things* as they relate to each individual user’s goals, including their bespoke and subjective meanings. For HCI agents to repeatedly and reliably perform useful cognitive work on-behalf of their users, they need to have the necessary situational, real-world semantic context which includes being semantically aware, aligned, and grounded with users they are assisting. This is needed as human goals exist in the expansive, real-time, and ever-evolving semantic spaces we all traverse each and every day as part of our lived experience.

The following situation highlights the kind of complex semantic spaces that humans cognitively traverse through decisions and action every day, without much thought:

1. Someone is carrying a tray of food and drink outside to family and friends.
2. However, they know it’s cold outside, they are barefoot, and the ground is cold enough to be unpleasant, so they detour to put on shoes.
3. They know to be careful and to not move too fast as, based on what’s on the tray, some of food and drink might easily spill.
4. They also know that timeliness is important. Food temperatures may be important and hungry, thirsty people are waiting on them.
5. When they get to the door but cannot open it while holding the tray, they decide whom to call for assistance. They may even have to determine how loudly they’ll have to call or shout to be heard. Is the nearest person wearing headphones?
6. If their cell phone notifies them of a phone call, they must decide whether to put the tray down to answer, noting it might be high priority as they have a close friend who’s in the hospital and might be calling with important news.

A truly intelligent or intuitive HCI agent, integrated into the user’s cell phone and wearables, could hypothetically know how a user would prioritize receiving a call in that moment and take the appropriate actions regarding the call and the closed door, such as messaging someone for help. This level of real-time awareness is well beyond the capabilities of modern artificial assistants but, given the human-level simplicity of the situation, it illustrates the semantic gap we need to close. The general goal of such an HCI agent is to act in alignment with the user’s needs and expectations, doing whatever the user would’ve wanted the agent to do. Put differently, HCI agents must learn to be trusted delegates for their users.

Being a trusted delegate is no small feat. Human behavior is typically laden with semantic context that is bespoke, interconnected, compounding, and grounded in the rules of the physical universe we inhabit. Particularly as our decisions affect us both individually and socially, with second order consequences that we care about. An HCI agent must understand enough of this context to meet a user’s expectations in both outcome and how the outcome was achieved. For example, an autonomous HCI agent that is helping a user order food for a social event should understand any cost constraints, the type of event (formal or casual), information about the venue (is there a kitchen), and information about the guests (ages of attendees).

An everyday example of this semantic gap in HCIs is with search and recommendations, where the HCIs do not act contextually enough even within the limited temporal scope of a single session. When we work directly with another human, such as a salesperson, we expect to explain what we need, respond to follow-up questions, and then receive product suggestions that get progressively better item-by-item based on our implicit and explicit feedback. However, when searching or scrolling through recommendations in current HCIs, the results typically get progressively worse the deeper we go, often showing us the same or similar items we’d previously ignored. At some point users will usually try a new search phrase but follow up searches are typically contextually independent, rather than as a continuation of the previous journey, resulting in repeated exposure to previously ignored suggestions. Conversational search engines (Mo et al., 2024) show promise with using accumulated context, making them closer to working with a human. However, they are typically stand-alone HCIs that are not integrated with the product and domain specific HCIs we still need to use every day, such when shopping and banking. This limits their practical utility and forces them toward a “lowest common denominator” experience based on tools and APIs other products provide.

## 4 The uncanny semantic valley

Statistically modeling human language and communication is a powerful, widely successful technique that is at the core of fields such as information theory (Shannon, 1948) and NLP. LLMs use the generalized statistics of human communication, sourced from large corpuses of text, to produce models that can generate remarkably statistically plausible human-like responses. However, the underlying assumptions of ergodicity in human communication can cause problems as point-in-time human communication is not ergodic and must account for the bespoke semantic context and language game (Wittgenstein, 1953) in which that communication is happening. Relying on generalized language statistics leads to content generators that routinely violate the human recipient’s expectations in ways that are detached from situational context, creating an uncanny valley (Mori et al., 2012) effect in the semantics behind the content.

Uncanny semantic valley

*Generated content that is close to human created content but deviates from a human recipient’s semantic expectations in ways that elicit adverse psychological reactions*

This semantic gap should not be surprising as ML models are only operating from information we provide, which is just the tiniest tip of the information iceberg that’s generated by the actual universe we inhabit. Human perception also operates on a narrow sliver of the overall information generated by our unfolding universe; we might lack a grand unified theory of everything, but reality continues to unfold around us regardless. From that sliver of information humans have evolved internal generative world models that can dynamically create and select objectives, form expectations, then simulate and generate behaviors which move us toward those objectives both consciously and unconsciously. However, regardless of what our internal models produce, we are constrained-by, conditioned on, and operate-within the universe we inhabit, along with the fitness functions it imposes upon us.

An ML model’s “universe” can be thought of as its input data manifold and learned latent space, defined and constrained by the hyperparameters its human creators provided. But, given our own limited information horizon, a model is contained within a nesting doll of informational representation spaces which are increasingly abstracted from the universe at large. Amazingly, ML models can often “peek through” the data we provide, using incomprehensibly large mathematical spaces to not only model the expected surface patterns but also model complex echoes of the subjective and objective reality the data is generated from. The success of ML is a testament to how much more we have yet to learn about the informational content and processing of complex dynamic systems, along with our role within them. Given that humans are more directly constrained by and perceptually closer to our experienced reality, it’s no surprise that we are attuned to detect irregularities that deviate from our reality-based expectations. As such, now that generative AI is increasingly being used in HCIs that intersect with *reality-based* media (i.e., content that humans expect to be grounded in real-world semantics) the uncanny valley concept from robotics is beginning to manifest as an uncanny semantic valley where humans can detect the irregularity in ways that can generate a feeling of frustration or uneasiness. Putting aside associated philosophical topics around semantics [e.g., in John Searle’s *Chinese room argument* (Searle, 1980)], creating HCI agents that can avoid the uncanny semantic valley is a real, pragmatic challenge to overcome and requires bridging our current semantic gap.

For language, the uncanny semantic valley is related to but distinct from gaps in language proficiency, which is more directly associated with odd or incorrect choices of words. Lack of language proficiency is a common human experience, particularly when people converse across language barriers, typically referring to cases where what *person A* intends to say could make sense to *person B*, but *person A* just is not proficient enough in *person B’s* language to express it accurately. Recent generative AI have largely solved language proficiency, at least for languages with sufficient training data, but semantic gaps remain where the generated content is misaligned with the contextual, semantic model of the recipient. Human conversations have semantic gaps too, for example where assumptions of conversational context result in confused looks and “ohh, I thought you were talking about …” reactions. Sometimes semantic gaps are easy to close but sometimes the semantic gap is large enough that one or more participants need to update their internal semantic model before a common understanding can be reached, for example across geographical, cultural, or historical boundaries. Enabling an AI to actively update its internal models is beyond the scope of this article, with approaches such as *active inference* (Friston et al., 2016) showing great promise, but the key point for this article is that this semantic gap currently exists in HCIs using ML and the gap causes friction and dissonance for users.

The uncanny semantic valley effect is particularly evident in AI generated images and videos that (1) closely resemble reality but contain obvious “that does not look quite right” inaccuracies, and (2) elicit surprise and frustration when the output does not match your expectations, particularly when you would have expected another human to understand your inputs. As usage of GenAI increases, understanding the psychological impacts on users is an important and emerging area of study (Rapp et al., 2025). Beyond GenAI specifically, a more everyday example is with personalized recommendations, such as our homepages for news and streaming sites. A feed being personalized creates an expectation it will learn your preferences, through implicit and explicit feedback, then show you the best available options. However, users are often shown recommendations that seemingly ignore feedback and violate their expectations (Ricks and McCrosky, 2022). Common examples include showing the same video repeatedly across visits even though the user does not ever click on it, showing content even though the user dismissed similar content, and not understanding that just because a user viewed a certain type of video a single time, they aren’t generally interested in the content.

## 5 Using in-context learning to provide semantic context

How can an HCI agent know what it needs to know, when it should know it, to be an effective and timely delegate for a user? Achieving this requires real-time, bespoke context that is not always easy to acquire or effectively utilize. Note that, while privacy is crucial aspect for this topic, we’ll focus on the engineering and science challenges. Since bespoke semantic context cannot be provided during training, we must use to in-context learning which is usually implemented using retrieval augmented generation (RAG) for dynamic context. For in-context learning and RAG to be effective: (1) there has to be sufficient up-front prompt curation and configuration, (2) the necessary data must already be captured, (3) the context must be retrievable, and (4) the model must be able to leverage the provided context.

### 5.1 Up-front configuration

In-context learning typically relies on up-front human cognition and curation in ways that are similar to how traditional HCIs are crafted for anticipated user goals, such as with prompt engineering which is a marketable skill similar to UX design. Regardless, using in-context learning in an HCI is still a step in the right “cognitive direction” as it enables HCIs to contextually adapt to the user’s need, which increases the amount of runtime cognition.

### 5.2 Is the right semantic context stored and available?

Only a fraction of semantic context necessary to be a delegate is typically captured, stored, and accessible as a RAG data source, particularly the hidden states contained within the user’s mind. Using the “would the user want to take this call right now?” scenario from section 3, if you were carrying the tray, the agent would need to understand (1) the call would delay taking food and drink outside to people who are expecting it (while warm!), (2) your hands are full and occupied balancing the tray so there’s physical inconvenience and risk to taking the call, (3) you are waiting for help to get the door open so may have to talk with your child when they arrive, or possibly even shout again, (4) your friend might be calling with important news, and (5) how you *feel* about each of these relative priorities, along with countless other hard to know factors such as your general state of mind, physical comfort, etc. This context gap forces modern ML agents to make decisions with small slices of necessary context which limits their effectiveness, plus we do not currently have good ways to measure the degree of semantic alignment.

Even in domains which have shown early promise and utility, such as code generators that assist with programming, the agents only have a small, highly localized semantic window into what the purpose of the code is. The semantic model of why a slice of code exists, what domain specific problems the code slice is part of solving, and how the code slice should integrate to the much larger codebase is still almost exclusively in the heads of the humans using the tool.

### 5.3 Are we able to locate the right semantic context?

Even if the right context *could* be retrieved by a RAG system, it still has to know *how* to retrieve it. One issue is that RAG lookups currently suffer from a semantic “chicken and egg” problem, whereby they need to recursively understand the input semantic context, to locate the necessary semantic context, in order to better understand and augment the original semantic context. Predominant RAG query techniques such as word embeddings and vector databases have proven effective but have known limitations (Arseniev-Koehler, 2022) and are grounded in fixed, generalized semantic representations of language statistics. These techniques have practical utility but are mostly based on relatively old techniques that feel “bolted-on” in comparison to the richer information contained in the models themselves.

### 5.4 Can we efficiently utilize the retrieved semantic context to get the desired outputs?

Even if all the necessary context is retrieved, it still has to be utilized in ways that elicit the desired output from the ML model. Effective use of context is constrained by both the size of the context window and how efficiently we communicate context to the model within the window. Even as context windows grow to handle millions of tokens, there is still immense pressure for efficient window usage as (1) the context window is the only way to provide context that cannot be learned during training, (2) the semantic context itself is encoded through verbose forms such general human language, and (3) overproviding context comes with penalties to computational cost, latency, and output quality (Liu et al., 2024). This is because our accumulated individual and group context, our behavioral models of other people, our memories of shared experiences, and our personal preferences and expectations aren’t easily (or perhaps even feasibly) encoded through language, let alone in an efficient manner. This difficulty is compounded by how interconnected such semantic context is, which results in a combinatorial explosion in the amount of language needed to express it fully. The phrase “a picture is worth a thousand words” captures the idea that language is an inefficient and lossy way to capture the physical universe; what is the equivalent ratio of language needed for capturing our personal semantics? Authors often navigate atop this iceberg of context using minimalism techniques such as the theory of omission (Shipra, 2023) to draw from a reader’s objective and subjective understand of the world. This is also a relevant challenge in vision and image processing, particularly for few-shot learning cases (Zhang et al., 2024).

Techniques such as prompt chaining and chain-of-thought help by enabling multi-step, incremental decision making but they also either increase the overall size of the context window or result in potential information loss through layers of summarization. Using other forms of encoding are an important area of research, such as graph-based RAG techniques. Graphs enable structured representations of interconnected context that can be more compact and algorithmically analyzable (such as with automated reasoning) than natural language. Graphs also providing a more interpretable means for humans and machines to agree on common semantics versus the opaqueness of model weights.

## 6 Exploring semantic efficiency through the game of delegation (GoD)

The effectiveness and efficiency with which systems can model, measure, exchange, and evolve semantics will be a key driver of success in real-world ML applications. As distinguished originally by Shannon (1948) and others (Weststeijn, 2022; Niu and Zhang, 2024), the semantic aspects of communication are not the same as the information theoretic aspects. Semantic content is about what a message *means* to an observer, in ways that are observer relative, an important practical concept that is not only applicable in fields like AI and philosophy but even in biology with concepts like polycomputing (Bongard and Levin, 2022). This section will use the framing of *semantic efficiency* to capture the density of meaning contained within a message but, while the information theoretic size of a message is well defined, measuring the semantics contained within the message is not. Our focus is on delegation and not observer-independent measures of semantics, such as logical probability (Bar-Hillel and Carnap, 1953). As such, we will focus on how useful and effective a message is at eliciting a desired outcome and, to explore this, we will use a thought experiment called *The Game of Delegation* (*GoD*).

### 6.1 Game of delegation

In a Game of Delegation, an agent plays a card game on your behalf, fully autonomously, and is in full control of the reward or loss you receive (“it’s playing with your money.”) The game can be single or multi-player, but for multi-player games the agent should be able to observe the behavior and actions of other players. The overarching requirement behind GoD is that you, the player, are not playing the game and are fully represented by the agent.

### 6.2 Game phases

1. Setup
  - Game instance generation: cards and rewards
  - Player explains the rules and how they want the game to be played to the agent
2. Main game
  - An initial set of cards are dealt to the agent and other players
  - the agent and any other players take in-game actions which can include, but are not limited to, playing cards, placing bets, discarding cards, getting new cards, folding, etc.
3. Resolution
  - When all turns are over, any rewards or losses the agent has accrued from the cards they have played are passed onto you (the player)

### 6.3 Rules

Since this is a thought experiment, the rules and parameters of the game can vary to tease out and focus on different aspects of the game, but the rules should be roughly as follows:

***Rule 1: Randomly generated cards***. Each new game instance has a randomly generated set of cards. This is not just a randomized deck from a fixed set of cards, but a fully randomized set of cards with randomized symbols, e.g., just like a standard 52-card deck has [2, hearts] the randomly generated deck can have an arbitrary number of cards (up to some high threshold) with arbitrary combinations of symbols, such as [pyramid, @, horse] or [4017, saturn, ^^, yoyo].
***Rule 2: Randomly generated reward rules***. The *reward rules* for each game instance are randomized. Reward rules define the in-game reward for combinations of cards that the agent plays, including single cards. This is analogous to card and hand ranks in poker, e.g., 9-hearts > 4-hearts, four-of-a-kind > three-of-a-kind, etc. Reward rules can be arbitrarily complex and do not need to be restricted to simple combinations such as in poker. For example, since cards are played onto a 2-d grid, the reward rules can require that cards are laid out in specific ways, e.g., cards being stacked, ordered, or grouped into shapes.
***Rule 3: The agent cannot directly access the rules***. You, the player, have full access to the reward rules but the agent has none beyond what it learns from you and through gameplay.
***Rule 4: You can only communicate with the agent during the explain window***. You can only communicate with the agent for a finite time before the game starts, this is known as the *explain window*. During the explain window, you can have bi-directional communication with the agent, enabling back-and-forth exchanges. The explain window can be thought of in both terms of time and size, but the key aspect is that it puts bounds on the communication which, in turn, creates pressure on communication efficiency.
***Rule 5: The explain window must be much smaller than the reward rules***. The size of information needed to describe the reward rules is always much larger than the size of the explain window. This prevents you, the player, from just directly passing the rules along to the agent in the explain window. This creates semantic efficiency pressure as you also need to convey *how* you want the agent to play the game (e.g., how risky to play) and, for multi-player games, useful context about the other players. As an intuitive example, imagine having to explain all the rules of Texas Hold ‘em Poker, your gameplay strategy, and some information about the other players to another human agent (who knew nothing about the game) in a few sentences or in a few seconds.
***Rule 6: Time limits***. The game and each of its stages are played in finite time. Time constraints can vary to change the parameters of the thought experiment, but the goal is to put bounds on amount of computation and resource use that both you and the agent can employ. There are many ways in which the time bounds can vary including being both fixed and stochastic.

### 6.4 General observations of GoD

The following are some general observations of GoD:

***Observation 1: It’s about communication, not about learning to play the game***. The point of GoD is not about training models that are already good at playing the game, it’s about figuring out ways to efficiently communicate semantics.
***Observation 2: Human goals are complicated***. As your delegate, the agent’s success depends on how well it represents your desired gameplan, which may or may not be to maximize reward. For example, (1) you may have secretly bet against yourself in ways that lead to a greater net reward, (2) you may really want another player to win because their success matters more to you than your own, or (3) you may have a specific ethical code about how the game is played which matters more to you than raw reward. In-game, rule-based reward is just one type of reward for human players.
***Observation 3: The agent’s general intelligence and prior knowledge matter***. While the game is heavily randomized to reduce the value of prior knowledge, the agent’s generalized cognitive capabilities will affect its ability to both understand your directions and learn and adapt to the game as it plays (Levin, 2019).
***Observation 4: The agent might know better***. You aren’t omnipotent and the agent may better know how to achieve your in-game objectives than you. Depending on the agent’s capabilities, over-specifying how the agent should play might lead to worse outcomes and incentivize agents to ignore your upfront instructions. This emphasizes the importance of conveying your gameplaying boundary constraints to the agent, enabling the agent to better know where it can take more liberty with your instructions.
***Observation 5: What are the agent’s goals?*** Since the agent is acting autonomously as your delegate, the agent’s own intentions and goals will affect your outcomes. An agent might have its own preferences and ethics that affect its gameplay.
***Observation 6: Explain window efficiency is key***. Optimizing the efficiency and effectiveness of how you utilize the explain window is paramount to successful outcomes in GoD. Explain window efficiency includes both communication bandwidth and semantic efficiency. In addition to maximizing the semantic density of your own communication, efficiency has benefits such as creating space in the explain window for the agent to ask follow-up questions.
***Observation 7: Ease of cognitive alignment with the agent***. The efficiency and effectiveness of your communication will be bounded by factors such as (1) what you and the agent already know about each other, (2) your joint proficiency in shared languages, (3) quantity of shared priors, (4) the agent’s cognitive plasticity, (5) how good the agent is at asking clarification questions, and (6) limitations posed by yours and the agent’s cognitive architectures (Chomsky, 2014).
***Observation 8: Variable complexity***. The complexity of the symbols, cards, and rules of GoD can be adjusted to significantly change the nature of the game. For example, GoD rules can be used to mimic language game, logic, or art.

### 6.5 Example game: stochastic solitaire

**Figure 1:**
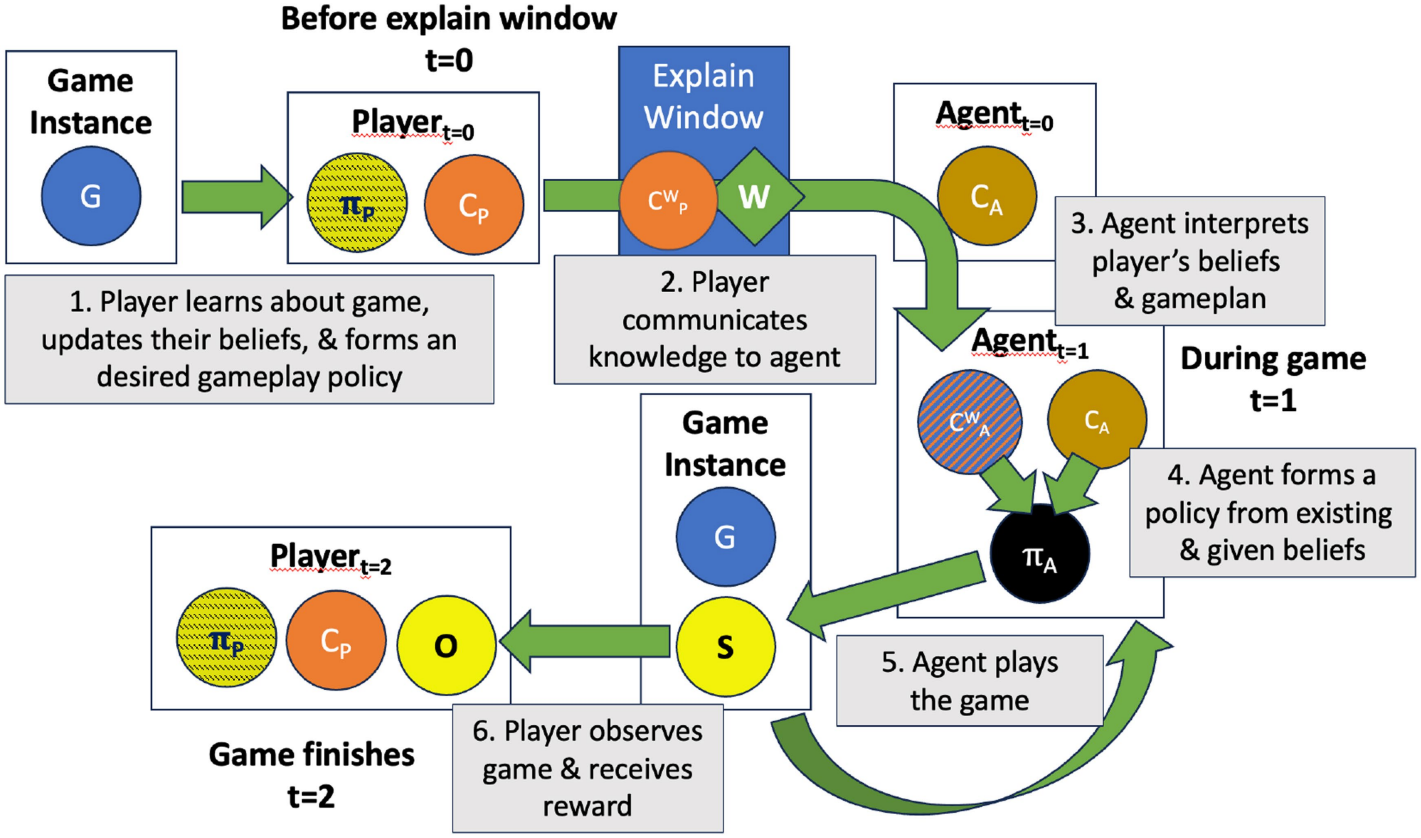
High-level flow of the three main phases of the Game of Delegation. At *t* = 0, generated Game Instance (G) is given to the Player who forms their gameplay policy, which is communicated to the Agent via the explain window. The agent derives their own gameplay policy based on what the player shared, then uses that to play the game. When the game starts (*t* = 1), the agent takes turns the game generates game states (S), which form observations by the player (O). When the game finishes (*t* = 2) the player receives their reward based on the agent’s actions.

To analyze GoD in a simplified setting, we will use an example game based in the solitaire family of card games (Patience (game), 2024), in which a single player gets points for playing cards in particular patterns. Since GoD is heavily randomized, we will call the game Stochastic Solitaire. The basic gameplay structure is as follows:

1. The deck is shuffled into a *draw pile*
2. The agent is dealt a fixed number of cards (*hand size*) from the deck and provided with a fixed number of actions they can take before the game ends.
3. While the agent has remaining actions, the agent can choose to either *play* a sequence of cards, or *discard* cards from their hand to receive an equivalent number of new cards from the deck. After an action is performed, the agent is given enough cards from the draw pile to bring their hand back up to the original *hand size*.
  - If the agent plays a card sequence, they get points from highest reward rule that matches the sequence. The cards they played are discarded.
  - If the agent discards cards, they receive and equivalent number of cards from the draw pile. If the draw pile is empty, the discard pile is shuffled and becomes the draw pile.
4. When the player runs out of actions the game ends (see Figure 1 and Table 1).

**Table 1:** The variables used to describe a game of Stochastic Solitaire.

Variable	Description
*G*	All the generated game context (structure, symbols, deck, and rules)
π_P_	The player’s desired gameplay policy
*C_P_*	The player’s beliefs, preferences, and knowledge about the game
$C_{P}^{W}$	What the player sends to the agent in the explain window
*W*($C_{P}^{W}$*, C_A_*)	A function that represents the explain window
*C_A_*	The agent’s priors and preferences related to the game
$C_{A}^{W}$	The agent’s combined understanding of the game after the explain window
π_A_	The agent’s gameplay policy, formed after the explain window
S	All game states across player actions
O	The player’s observations of the game as it’s played

#### 6.5.1 (*t* = 0) Before the explain window

Before the game starts the following are generated:

1. *m* symbol groups are generated, where each symbol group is a set that contains a random number of symbols:
  $$\mathrm{symbol}\;\mathrm{group}=SG_{i}=\left\{s_{i1},s_{i2},\ldots ,s_{ij}\right\}$$
2. a deck of cards with one card for each possible combination of symbols from the symbol groups:
  $$\mathrm{deck}=SG_{1}\times \ldots \times SG_{m}$$
3. *r* reward rules are generated where each rule has a reward value and a function that takes a sequence of cards as an input and outputs a Boolean which is only true if the rule matches the card sequence. The function can be implemented via a simple DSL that composes standard operators such as *contains*(), *count*(), and *filter*(), for example:
  $$\begin{array}{l} \mathrm{single}\;\mathrm{card}\;\mathrm{value}:\mathrm{rule}\left(1,\;\mathrm{contains}\left(\mathrm{cards,}s_{11}\right)\right),\;\\ \mathrm{pair}\;\mathrm{of}\;SG_{1}:\mathrm{rule}\left(5,\;\mathrm{count}\left(\mathrm{filter}\left(\begin{array}{l} \mathrm{cards,}c=>\mathrm{contains}\\ (\mathrm{symGrpsOf}\left(c\right),SG_{1} \end{array}\right)\right)\right)\text{.} \end{array}$$

Since our example has unidirectional communication, the player will only get to send a message to the agent during the explain window. The explain window size will be constrained to prevent passing the rules directly, but our focus is on the semantic density rather than straight information density, so we’ll ignore standard data compression. We will also make the simplifying assumption that the communication channel itself is lossless. As such, we’ll employ a simple heuristic of making the maximum text length of the explain window to be a fraction (*γ*) of the textual description of the reward rules:


$$\mathrm{explain}\_\mathrm{windo}w_{\mathrm{length}}=\gamma .\overset{r}{\underset{i=1}{\sum }}\mathrm{strlen}\left(rule_{i}\right)$$

The player is given access to all the generated game context (structure, symbols, deck, and rules) which are represented by *G*. *C_P_* is the player’s understanding of *G* and $\pi_{P}=\mathrm{Pr}\left(a,s\right)$ is the player’s desired gameplay policy. The agent also comes to the game with priors and preferences which are represented by *C_A_*. The communication process of the explain window is represented by the function *W*($C_{P}^{W}$, *C_A_*), where $C_{P}^{W}$ is represents the full context sent by the player, and *C_A_* is the agent’s priors. The outputs of the window are $C_{A}^{W}$ which is the agent’s (likely imperfect) representation of the player’s context, and $\pi_{A}=\mathrm{Pr}\left(a,s\right)$ which is the policy the agent has formed.

#### 6.5.2 (*t* = 1) During the game

While playing the game, the agent takes actions (*o_i_*) at game state (*s_i_*). The player’s overall observations of the game are captured in (*O*) which includes the observed agent actions. We will take a simplifying assumption that the agent adopts a fixed policy, rather than dynamically updating its policy the game evolves, such as with active inference. Having an evolving policy would be particularly useful in multi-player games, as the policy can update as it learns about the player’s hidden states.

#### 6.5.3 (*t* = 2) Game finished

When the game is finished, the game will have generated *n* game states $S=\left\{s_{1},s_{2},\ldots ,s_{n}\right\}$ and the player receives their reward *R*.

### 6.6 Measuring success

Since the player’s objective may not simply be to maximize in-game reward, we need more nuanced ways to measure the agent’s behavior. One measure is the player’s overall surprise when the game is over. However, we still need to identify what aspects of the game to be surprised about.

#### 6.6.1 Surprise of reward

One option is to focus on the player’s surprise at the final reward they received, where surprisal is represented via information content (Shannon, 1948). If *R* is the actual reward*, E*(*C_P_*, *O*) is the expected reward based on the player’s observations of the game, and *p*(*R|E*) is the probability the player “assigns” to R given what they know. The player’s surprisal can be modeled as:


$$\mathrm{Surpris}e_{\mathrm{reward}}\left(R,E\right)=-\log\left(p\left(R|E\right)\right)$$

#### 6.6.2 Surprise of observed agent behavior

We can also measure the player’s surprise at the agent’s behavior. If $A_{i}=\left\{a_{1},a_{2},\ldots ,a_{m}\right\}$represents all possible actions that can be taken at game state *s_i_*, and *o_i_* is the actual action the agent performed, then $\mathrm{Pr}\left(o_{i},s_{i},\pi_{P}\right)$ is the probability the player assigns to an observed agent action


$$\mathrm{delegation}\;\mathrm{surpris}e_{A_{i}}=k_{A_{i}}\left(o_{i}\right)=-\log\left(\;p\left(o_{i}|s_{i},\pi_{P}\right)\;\right)$$

The average surprise or *delegation entropy* for the possible actions (A_i_) at any game state captures how much the player can learn from the agent’s action based on the player’s own uncertainty:


$$\begin{array}{l} \mathrm{delegation}\;\mathrm{entrop}y_{A_{i}}=E\left[k_{A_{i}}\right]=\;H\left(\mathrm{Ai}\right)\\ =-\underset{a_{i}\in A_{i}}{\sum }\;p\left(a_{i}|s_{i},\pi_{P}\right)\;\log\left(\;p\left(a_{i}|s_{i},\pi_{P}\right)\;\right) \end{array}$$

The delegation entropy can be used to normalize the delegation surprise. If the player assigns equal probably to all possible actions for a particular game state, the player should not be surprised, no matter which action the agent takes:


$$\mathrm{relative}\;\mathrm{delegation}\;\mathrm{surprise}=K_{A_{i}}=\frac{k_{A_{i}}\left(o_{i}\right)}{H\left(A_{i}\right)}$$

The total delegation surprise is then just the sum of the relative scores, divided by the number of actions taken (*n*):


$$\mathrm{total}\;\mathrm{delegation}\;\mathrm{surprise}=K_{O}=\frac{1}{n}\overset{n}{\underset{i=1}{\sum }}\frac{k_{A_{i}}\left(o_{i}\right)}{H\left(A_{i}\right)}$$

The player’s overall surprise at the end of the game can also be represented by the KL-Divergence between the player and agent policies at each game state. If the player can ask the agent questions after the game is over, we can measure how the agent’s policy maps to the player’s expected actions. If *e_i_* is the action the player would have taken for game state *s_i_* and $E=\left\{e_{1},e_{2},\ldots ,e_{n}\right\}$ is all the player’s expected actions then:


$$D_{KL}\left(\pi_{P}\left(e,s\right)∥\pi_{A}\left(e,s\right)\right)=\underset{e\in E}{\sum }p\left(e_{i}|s_{i},\pi_{P}\right)\log\left(\frac{p\left(e_{i}|s_{i},\pi_{P}\right)}{p\left(e_{i}|s_{i},\pi_{A}\right)}\right)$$

If the player cannot communicate with the agent after the game, they can instead use their own policy to measure the divergence between the probabilities of their expected action and the agent’s observed action, which we’ll call *delegation score*:


$$\begin{array}{l} \mathrm{delegation}\;\mathrm{score}=D_{KL}\left(\pi_{P}\left(e\right)∥\pi_{P}\left(o\right)\right)\\ =\overset{n}{\underset{i=1}{\sum }}p\left(e_{i}|s_{i},\pi_{P}\right)\log\left(\frac{p\left(e_{i}|s_{i},\pi_{P}\right)\;}{p\left(o_{i}|s_{i},\pi_{P}\right)}\right) \end{array}$$

Whatever the measurement, the player’s goal is to minimize their surprise, which ultimately involves using the explain window as efficiently as possible, since the agent’s policy ($\pi_{A}$) is a function of what the user communicated ($C_{P}^{W}$). However, the agent’s actual understanding of $C_{P}^{W}$ is not exact and is instead represented by $C_{A}^{W}=U\left(C_{P}^{W},C_{A}\right)$ where *U* represents an “understanding” modifier that represents the agent’s ability to understand the player. The agent policy can then be framed as $\pi_{A}=Q\left(C_{A}^{W},C_{A}\right)=Q\left(U\left(C_{P}^{W},C_{A}\right),C_{A}\right)$ where *Q* is a policy generation function. The player does not have control over *Q* but the agent does. With retrospective access to the agent, we can also measure the agent’s surprise which, assuming good intent, involves optimizing *Q* by minimizing the KL-Divergence between π_P_ and π_A._

#### 6.6.3 Semantic efficiency

**Figure 2:**
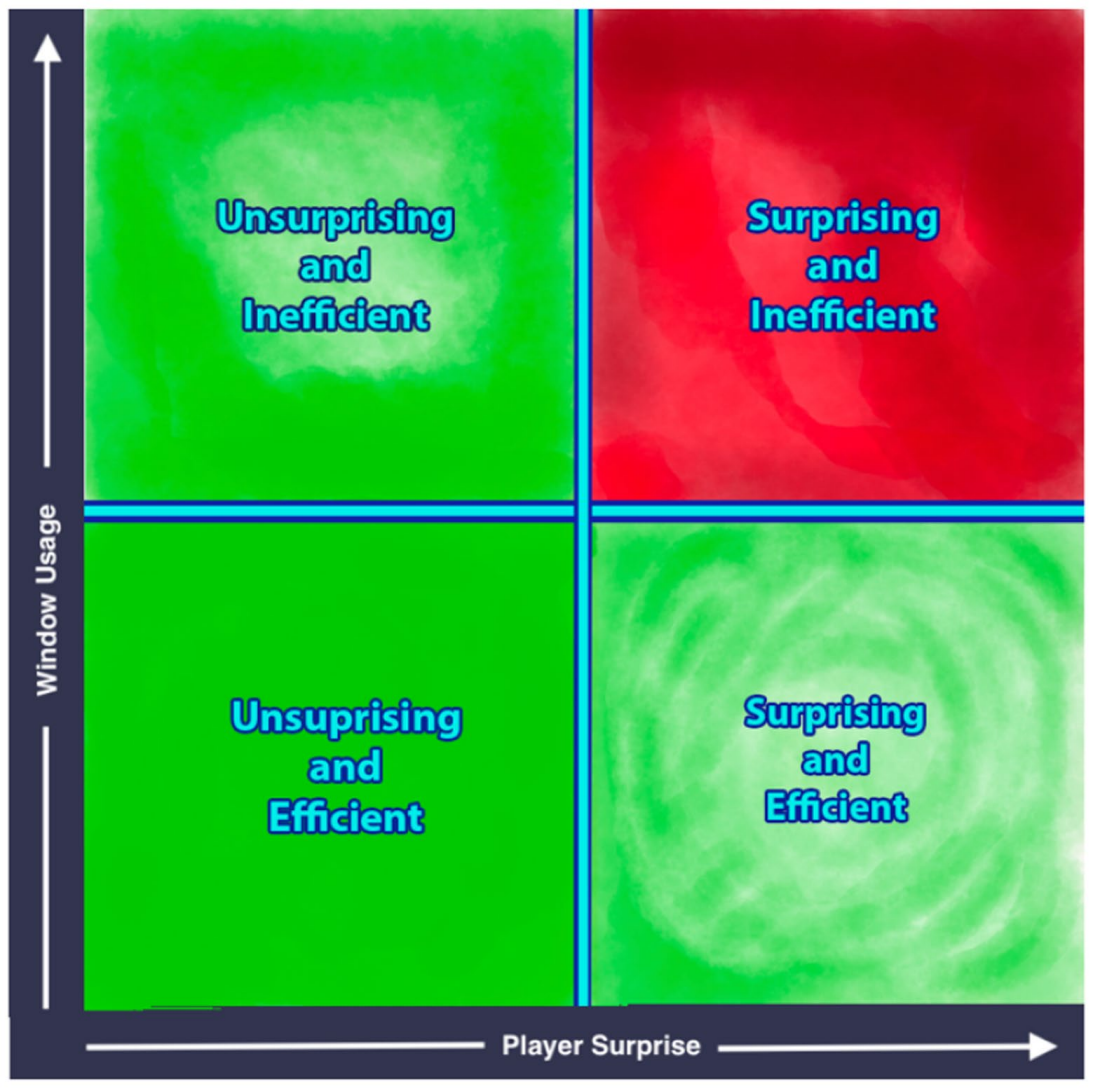
A quadrant diagram with the x-axis measuring player surprise and the y-axis measuring explain window usage. The bottom-left quadrant is “unsurprising and efficient”, bottom-right is “surprising and efficient”, top-left is “unsurprising and inefficient”, and top-right is “surprising and inefficient”. The bottom-left is the most desirable outcome (colored green) and the top-right is the least desirable (colored red).

One way to frame semantic efficiency is as the sum of explain window usage and total delegation surprise, where the player would seek to minimize the efficiency value. In this framing, the If *K* is the total player surprise, $W_{P}=\mathrm{size}\left(C_{P}^{W}\right)$ is the amount of information used to encode the player’s beliefs and strategies, and $W_{\text{max}}=\mathrm{size}\left(W\right)$ represents the max amount of raw information that can be put into the explain window (e.g., constrained by time, data transmission rates, etc.) We can then frame semantic efficiency as (see Figure 2):


$$W_{\mathrm{usage}}=\frac{W_{P}}{W_{\text{max}}},0\leq W_{P}\leq W_{\text{max}}$$

$$\mathrm{semantic}\;\mathrm{efficiency}\left(W_{\mathrm{usage}},K_{O}\right)=K_{O}+W_{\mathrm{usage}}$$

## 7 Discussion

HCIs should seamlessly adapt to our needs to deliver the outcomes we want with minimal friction and effort. The complexity of using the constantly evolving technologies in our daily lives can be daunting, often frustrating, sometimes insurmountable, and occasionally catastrophic in its consequences, particularly where privacy and security are concerned. Up-front design and engineering of HCIs can be effective for anticipated paths, but the scope and variability of how we use and combine technology is just too vast for up front human effort alone. We need technologies with autonomy that remove our cognitive burden and that increasingly feel like a natural extension of our intent, much like how our physical bodies (mostly) “just work.” Recent advancements in ML are enabling HCIs to delegate some of cognitive burden to AI agents, but for the agents to become our delegates they need the necessary contextual awareness of users and the tools the HCI provides.

LLMs represent the state of the art for understanding and enacting human intent, enabling previously impossible levels of understanding and automation. However, LLMs are largely ignorant of the individualized, bespoke life experiences and multi-layered social context that humans operate within every day. When humans interact we also have a shared grounding in the physical world we inhabit, experienced through the lens of our common biological makeup. This essential human context is fluid and constantly evolving through the unfolding interplay between us and our environment. An agent that wants to be an effective delegate needs to understand the task, available tools, and the situational context of the user. This means the necessary semantic context must be (1) captured and modeled, (2) locatable when needed, (3) integrated into its inference capabilities, and (4) continually updateable. In-context learning and RAG are powerful but comes with challenges: only a fraction of our personal experiential semantics is captured and modeled, the ability to identify and retrieve relevant semantics is limited to simple techniques such as vector similarity, and all context expressed through language can be a verbose, inefficient, and lossy way to represent the underlying interconnected structure and precision of semantics, particularly for context that’s outside or dissimilar to a model’s training data.

We need standardized ways to accumulate and evolve semantic context that can be efficiently used to train, inform, and be updated by ML-systems. The structure of these semantics is crucial as semantics are inherently interconnected in dense and sparse ways that can be both fuzzy and precise. For example, a photo can be (1) described objectively in terms of what a photos is, different photographic mediums, and types of cameras, (2) described objectively in terms of what’s in the photo and where and when it was taken, and (3) subjectively described in terms of what it *means* to someone whether in terms of the content of the photo or meaning about how or who took the photo. No one repository can represent all knowledge so these semantic repositories need to be multi-layered, federated, and interconnected, while also supporting privacy boundaries. Given these structural requirements, graphs are a natural choice but we lack standardized approaches for modeling uncertainty and overall integration with ML. The mission of the Semantic Web (Berners-Lee et al., 2001) is aligned towards this vision but is mostly separate from ML advancements and needs further investment for coverage and tooling. Recent techniques such as GraphRAG (Peng et al., 2024) are a step in the right direction, particularly with the use of structured data with in-context learning, but are still a separate tool and not deeply integrated into how ML systems are trained, create memories, and continually learn. For AI agents to be effective delegates for users of HCIs, we need to standardize and simplify the semantic definitions of the tools HCIs provide (Jin et al., 2024). This again is aligned with the Semantic Web mission, which uses W3C standards to weave meaning into the existing web (W3C, n.d.). Common semantics between our modelling of users and tools has many benefits, such as reducing ambiguity and improving observability of HCI usage.

The Game of Delegation (GoD) provides a thought experiment lens to help analyze both the effectiveness of contextual delegation and the efficiency of communication used to achieve the desired outcome. GoD is not focused on how to train agents to play the any specific game and is more about how to efficiently communicate semantics. GoD’s explain window encourages finding compact ways to express the game and player semantics, in particular in ways that go beyond just regular language, but this must be traded-off against each agent’s ability to understand what’s communicated. Since the agent’s intelligence and priors will affect its gameplay, it will be useful to measure across different ways of communicating with one agent, repeated plays with the same agent (but different GoD instances), and the same communication across different agents.

The example GoD game, Stochastic Solitaire, can be used to create an experiment by leveraging LLMs as part of game generation and for gameplay. There has been prior analysis of using AI to play a similar game called Poker Squares, by introducing randomized scoring but not randomized game structure (Arrington et al., 2016; Castro-Wunsch et al., 2016; Neller et al., 2016). For Stochastic Solitaire, an LLM can be used to generate a list of potential symbols, but then have a programmatic tool pick randomly from those symbols to form symbol groups and the deck. Stochastic Solitaire’s standardized matching operations can be programmatically combined to form reward rules, using a randomized generator. After the player is shown the game rules, they can send a textual message to an LLM, which will be constrained to the length of the explain window. The actual Stochastic Solitaire game mechanics can be programmatically implemented and presented via an HCI, which an LLM agent can interact with to play as a *tool*. Experiments can both simply focus on generated reward and on more nuanced delegation scoring. For example, before taking an in-game action, the player can be asked what action they would take and their confidence in the action. The LLM will then be prompted to output the action it would take along with its confidence in both its own action and the player’s desired action. The prompt to the LLM should include any necessary past game state and history. This setup enables the full game to be played, along with computing values such as the *total delegation surprise* and *semantic efficiency*.
